# Improving on legacy conferences by moving online

**DOI:** 10.7554/eLife.57892

**Published:** 2020-04-20

**Authors:** Titipat Achakulvisut, Tulakan Ruangrong, Isil Bilgin, Sofie Van Den Bossche, Brad Wyble, Dan FM Goodman, Konrad P Kording

**Affiliations:** 1 Department of Bioengineering, University of PennsylvaniaPhiladelphiaUnited States; 2 Department of Biomedical Engineering, Mahidol UniversityNakhon PathomThailand; 3 School of Biological Sciences, University of ReadingReadingUnited Kingdom; 4 Department of Data Analysis, Ghent UniversityGhentBelgium; 5 Department of Psychology, Penn State UniversityUniversity ParkUnited States; 6 Department of Electrical and Electronic Engineering, Imperial College LondonLondonUnited Kingdom; 7 Department of Bioengineering and the Department of Psychology, University of PennsylvaniaPhiladelphiaUnited States

**Keywords:** scientific meetings and conferences, scientific community, research culture, collaboration, neuroscience, climate change

## Abstract

Scientific conferences and meetings have an important role in research, but they also suffer from a number of disadvantages: in particular, they can have a massive carbon footprint, they are time-consuming, and the high costs involved in attending can exclude many potential participants. The COVID-19 pandemic has led to the cancellation of many conferences, forcing the scientific community to explore online alternatives. Here, we report on our experiences of organizing an online neuroscience conference, neuromatch, that attracted some 3000 participants and featured two days of talks, debates, panel discussions, and one-on-one meetings facilitated by a matching algorithm. By offering most of the benefits of traditional conferences, several clear advantages, and with fewer of the downsides, we feel that online conferences have the potential to replace many legacy conferences.

## Introduction

Conferences are places where scientists go to meet other scientists and discuss the latest developments in their field. Talks are given, posters are presented, questions are asked (and sometimes answered), debates are held, collaborations are discussed, job opportunities are discovered, and friendships are renewed. Indeed, conferences and meetings are so central to scientific progress that many scientific societies expend enormous time and effort on the organization of annual meetings. Traditional conferences – or legacy conferences as we will call them in this article – clearly matter for scientific communities.

At the same time, by requiring our presence at a particular location, conferences take a lot from us. We have to interrupt our lives and leave our families to travel to a faraway place, and in doing so we produce significant carbon emissions (Nathans and Sterling, 2016; Pardee, 2015; Quinton, 2020) and waste (Kier-Byfield, 2019). Attending a legacy conference can consume an entire week of our lives, and often involves two days of travel. Moreover, going to a meeting requires us to pay for flights, taxis, accommodation, registration, and childcare at home, making conferences expensive for the scientific system, for university administration, and for scientists as individuals. Travel can also hinder scientists who might not make it due to lack of funding, travel bans (Hu, 2018), or visas.

And even when we all make it, we might be disappointed as conferences often do not live up to their promise: we may not be able to see the speaker in the big lecture hall, we may miss the colleague we were looking forward to meeting, or we may not meet the right people. Many good connections fail to be made simply due to the variability and constraints of physical places. Legacy conferences also suffer from major communication problems. Questions are typically asked in a first-come, first-served manner, or worse, by the loudest voices. Moreover, the physical dimensions of the venue place strong restrictions on the number of talks. In the way conferences are organized, they tend to reinforce current power structures. Thus, legacy conferences have considerable weaknesses which have, so far, been largely ignored.

The COVID-19 pandemic has led to most legacy conferences being cancelled or postponed (Viglione, 2020). With many scientists being forced to stay at home and many cancelled meetings, there is an acute need for online conferences. This produces a unique window of opportunity for innovating in the online conference space (Gichora et al., 2010; Abbott, 2020; Rolnick, 2019). Thanks to the current huge increase in attention, ongoing innovation in the virtual space is likely to dramatically improve online conferences, potentially making them a better overall experience than legacy conferences.

In this paper, we sketch the experiences that we had setting up neuromatch, an online neuroscience conference with 3000 participants (Kording, 2020). We partnered with the University of Pennsylvania and Imperial College, and two journals – *Neurons, Behavior, Data analysis and Theory* and *eLife*. The collaboration with eLife allowed us to incorporate a new component for early-career researchers (eLife, 2020; Sarabipour, 2020; Weissgerber et al., 2020). The conference was free for all participants. The running costs ($150 for a Crowdcast subscription and $60 for Amazon Web Services) were covered by lab start-up costs provided to one of us (KPK) by the University of Pennsylvania. Both in the run up and during the conference, we were forced to make rapid organizational changes, and some of these changes were clearly very helpful. We summarize our approach and discuss what worked and what did not work.

## Moving a conference online

Conferences work by providing participants with a broad set of communication and socialization channels. In this article we discuss how these channels can be established in an online conference.

### Poster sessions and short talks

At a legacy conference, posters allow for a larger number of scientists to present their work than would be possible with talks alone. For neuromatch, all abstracts were accepted for a short talk in a parallel session as there are no limitations on space for an online conference. In principle, these talks could be recorded, ensuring that nobody needs to miss any talk, although we did not have enough time to organize this for the first neuromatch. Short talk sessions featured three 12-minute talks, each followed by three minutes of questions, and there were up to six parallel tracks. We found that these short talks led to exciting debates that are typical for posters, and might allow more time for questions and debate at future events. Each parallel track was a separate Zoom room with a volunteer host, a system that mostly worked well except that some rooms were subject to persistent 'Zoombombing' (organized disruption by online miscreants), a problem that can be solved by careful management of the Zoom room settings, or by switching to other platforms such as Crowdcast (Kording, 2020).

### Invited and contributed talks

We largely retained the legacy conference format of a single track for invited talks (30 minutes plus 15 minutes for questions) from established scientists, and contributed talks (18 minutes plus 4 minutes for questions) selected from the submitted abstracts to highlight work from up-and-coming researchers. However, the online platform used – Crowdcast – allowed for some significant innovations. First, everyone was able to see the speaker more clearly than in a lecture theatre. Second, Crowdcast allows anyone to submit a question to ask the speaker at the end, and viewers can vote on those questions. This led to a question and answer session that was considerably more lively and democratic than in a typical legacy conference, where participants often note that the same established professors are asking the same questions at every talk. As in the case of the short talks, it may be better to extend the questions even more to capitalize on the quality of the questions asked in the safer and more democratic online format. The third innovation is the chat window that appears alongside the talk. We did not anticipate how significant this would be. Students and others were able to ask basic questions about definitions or ask for links to papers while the talk was going on. Other participants could answer them in real-time without disrupting the presentation, thereby allowing a deeper level of engagement by the audience than is possible in legacy conferences. Moreover, since recordings of these talks were available immediately after the session, it would be possible to go back and revisit portions of the talk that may have been missed or were presented too quickly.

### One-on-one meetings

One feature of a legacy conference that would appear to be impossible to replicate online is the social aspect: chance encounters during the coffee breaks, social events or banquets. In place of this aspect, neuromatch algorithmically matched attendees to other like-minded scientists for individual 15-minute chats. We use a combination of topic modeling techniques and linear programming to solve the matching problem based on a sample of their research abstracts (Achakulvisut et al., 2018). The matching part was based on a highly popular experiment carried out at the Conference on Cognitive Computational Neuroscience, but it is particularly well-suited to an online format. There remains considerable scope for further innovations in replicating or improving on the social experience of legacy conferences, especially as the online format may be less socially intimidating.

### Organizing is much easier

We managed to organize neuromatch extremely quickly with a small team and believe that online conferences can be much less burdensome to organize in general. There is no location to organize, no rooms to book, and no projectors, caterers, entertainment, travel or hotel reservations to worry about. Moreover, there is a worldwide pool of potential volunteers who can help remotely. Leading scientists are also, we feel, more likely to accept invitations to speak at online conferences because the time commitment is significantly less.

### Diversity and inclusivity

Legacy conferences come with additional challenges on the diversity and inclusivity of participants. For example, family duties, gender bias, disabilities, travel bans, limited funding, religious practices, and many other disadvantages may limit participation in legacy conferences. An online platform can remove many of these barriers to increase the potential set of individuals who can participate (Sarabipour, 2020). In neuromatch, we included a session devoted to early-career researchers and had an equal number of men and women among the invited speakers. Moreover, we accepted all short talk submissions.

## Attendance and engagement

Over 1200 people pre-registered, rising to 2000 registrations during the conference, and a total of 3000 registered viewers on Crowdcast. The largest group among the participants were graduate students (47.3%), followed by postdocs (19.9%), professors (11.7%), research staff and assistants (10.7%), researchers from industry (3.5%) and others (6.8%). We also had 468 attendees sign up for the one-on-one matching part of the conference (which had a much earlier deadline): 50% were primarily looking for new collaborations, 23% for casual conversation to spark ideas, and the remainder were potentially interested in both. Ensuring that participants are able to progress their career by meeting potential collaborators and learning about career opportunities should be a priority for anyone organizing an online conference.

Participants were very much engaged as reported by analytic tools that are unavailable in legacy conferences. At the peak, we had 912 attendees engage in the live session concurrently (with more than 100 simultaneous users on YouTube). We had a median of 678 live attendees per session, and an average live attendance of 4.2 sessions from a total of 21 main sessions. 38% of attendees rewatched talks a day after the conference.

## Conclusion

Neuromatch is an example of how an online conference can have a wide reach yet feel personal to those taking part. Indeed, many participants said that they preferred the online experience (including the social aspects) to a legacy conference. We hope that our experience will be helpful to anyone thinking of organizing an online conference, and that we are about to see the equivalent of a Cambrian explosion in the field of conferences. We are convinced that a shift from legacy to online conferences will make science better and be less harmful to the environment.
